# Supplementary material for: Recurrent issues with deep neural network models of visual recognition
Source: Sci Rep. 2025 Oct 17;15:36344. doi: 10.1038/s41598-025-20245-w (PMC12534488; doi:10.1038/s41598-025-20245-w)
Supplement: Supplementary file 1 — Supplementary Information. [file 41598_2025_20245_MOESM1_ESM.pdf]

Supplementary material for: *Recurrent issues with deep neural network models of visual recognition*

Maniquet, T., Op de Beeck, H., Costantino, A. (2024)

1 Models information

The following section contains extra information about the models included in the main paper. We provide some general information about all the models in table S1, followed by extra model-specific information for the six custom-made models (for other models from *CORnet*, *B* and *VGG*, see the respective original publications).

Model name	Source	Size (parameters)	Feedforward	Lateral	Feedback
CORnet_Z	Kubilius et al. (2018)	1,562,760	Yes	No	No
CORnet_V1_V1	Custom-made	1,599,880	Yes	L1-L1	No
CORnet_IT_IT	Custom-made	1,599,880	Yes	L4-L4	No
CORnet_RT	Kubilius et al. (2018)	4,700,040	Yes	Yes	No
CORnet_T	Custom-made	11,480,904	Yes	No	Yes
CORnet_LT	Custom-made	11,480,904	Yes	Yes	Yes
CORnet_ZD	Custom-made	10,973,640	Yes	No	No
CORnet_S	Kubilius et al. (2018)	52,907,720	Yes	Yes	No
B_net	Spoerer et al. (2017)	8,291,720	Yes	No	No
BL_net	Spoerer et al. (2017)	17,257,864	Yes	Yes	No
BT_net	Spoerer et al. (2017)	12,592,520	Yes	No	Yes
BD_net	Custom-made	25,699,720	Yes	No	No
BLT_net	Spoerer et al. (2017)	21,558,664	Yes	Yes	Yes
VGG11	Simonyan and Zisserman (2015)	9,426,696	Yes	No	No
VGG16	Simonyan and Zisserman (2015)	134,301,768	Yes	No	No

Table S1: General information on the models used.

C

Index	Name	Layer Type	Input Shape	Output Shape	Param #	Trainable
2	V1.conv	Conv2d	[1, 3, 224, 224]	[1, 64, 112, 112]	9472	True
3	V1.nonlin	ReLU	[1, 64, 112, 112]	[1, 64, 112, 112]	-	-
4	V1.pool	MaxPool2d	[1, 64, 112, 112]	[1, 64, 56, 56]	-	-
5	V1.output	Identity	[1, 64, 56, 56]	[1, 64, 56, 56]	-	-
7	V2.conv	Conv2d	[1, 64, 56, 56]	[1, 128, 56, 56]	73856	True
8	V2.nonlin	ReLU	[1, 128, 56, 56]	[1, 128, 56, 56]	-	-
9	V2.pool	MaxPool2d	[1, 128, 56, 56]	[1, 128, 28, 28]	-	-
10	V2.output	Identity	[1, 128, 28, 28]	[1, 128, 28, 28]	-	-
12	V4.conv	Conv2d	[1, 128, 28, 28]	[1, 256, 28, 28]	295168	True
13	V4.nonlin	ReLU	[1, 256, 28, 28]	[1, 256, 28, 28]	-	-
14	V4.pool	MaxPool2d	[1, 256, 28, 28]	[1, 256, 14, 14]	-	-
15	V4.output	Identity	[1, 256, 14, 14]	[1, 256, 14, 14]	-	-
17	IT.conv	Conv2d	[1, 256, 14, 14]	[1, 512, 14, 14]	1180160	True
18	IT.nonlin	ReLU	[1, 512, 14, 14]	[1, 512, 14, 14]	-	-
19	IT.pool	MaxPool2d	[1, 512, 14, 14]	[1, 512, 7, 7]	-	-
20	IT.output	Identity	[1, 512, 7, 7]	[1, 512, 7, 7]	-	-
22	decoder.avgpool	AdaptiveAvgPool2d	[1, 512, 7, 7]	[1, 512, 1, 1]	-	-
23	decoder.flatten	Flatten	[1, 512, 1, 1]	[1, 512]	-	-
24	decoder.linear	Linear	[1, 512]	[1, 8]	4104	True
25	decoder.output	Identity	[1, 8]	[1, 8]	-	-
Total params: 1562760						
Trainable params: 1562760						
Non-trainable params: 0						

Table S2: Layer specific information about **C** (CORnet Z).

C V1-V1

Index	Name	Layer Type	Input Shape	Output Shape	Param #	Trainable
2	V1.conv_input	Conv2d	[1, 3, 224, 224]	[1, 64, 56, 56]	9472	True
3	V1.norm_input	GroupNorm	[1, 64, 56, 56]	[1, 64, 56, 56]	128	True
4	V1.nonlin_input	ReLU	[1, 64, 56, 56]	[1, 64, 56, 56]	-	-
5	V1.conv1	Conv2d	[1, 64, 56, 56]	[1, 64, 56, 56]	36864	True
6	V1.norm1	GroupNorm	[1, 64, 56, 56]	[1, 64, 56, 56]	128	True
7	V1.nonlin1	ReLU	[1, 64, 56, 56]	[1, 64, 56, 56]	-	-
8	V1.output	Identity	[1, 64, 56, 56]	[1, 64, 56, 56]	-	-
10	V2.conv	Conv2d	[1, 64, 56, 56]	[1, 128, 56, 56]	73856	True
11	V2.nonlin	ReLU	[1, 128, 56, 56]	[1, 128, 56, 56]	-	-
12	V2.pool	MaxPool2d	[1, 128, 56, 56]	[1, 128, 28, 28]	-	-
13	V2.output	Identity	[1, 128, 28, 28]	[1, 128, 28, 28]	-	-
15	V4.conv	Conv2d	[1, 128, 28, 28]	[1, 256, 28, 28]	295168	True
16	V4.nonlin	ReLU	[1, 256, 28, 28]	[1, 256, 28, 28]	-	-
17	V4.pool	MaxPool2d	[1, 256, 28, 28]	[1, 256, 14, 14]	-	-
18	V4.output	Identity	[1, 256, 14, 14]	[1, 256, 14, 14]	-	-
20	IT.conv	Conv2d	[1, 256, 14, 14]	[1, 512, 14, 14]	1180160	True
21	IT.nonlin	ReLU	[1, 512, 14, 14]	[1, 512, 14, 14]	-	-
22	IT.pool	MaxPool2d	[1, 512, 14, 14]	[1, 512, 7, 7]	-	-
23	IT.output	Identity	[1, 512, 7, 7]	[1, 512, 7, 7]	-	-
25	decoder.avgpool	AdaptiveAvgPool2d	[1, 512, 7, 7]	[1, 512, 1, 1]	-	-
26	decoder.flatten	Flatten	[1, 512, 1, 1]	[1, 512]	-	-
27	decoder.linear	Linear	[1, 512]	[1, 8]	4104	True
Total params: 1599880						
Trainable params: 1599880						
Non-trainable params: 0						

Table S3: Layer specific information about **C V1-V1** (CORnet V1-V1).

C IT-IT

Index	Name	Layer Type	Input Shape	Output Shape	Param #	Trainable
2	V1.conv	Conv2d	[1, 3, 224, 224]	[1, 64, 56, 56]	9472	True
3	V1.nonlin	ReLU	[1, 64, 56, 56]	[1, 64, 56, 56]	-	-
4	V1.pool	MaxPool2d	[1, 64, 56, 56]	[1, 64, 28, 28]	-	-
5	V1.output	Identity	[1, 64, 28, 28]	[1, 64, 28, 28]	-	-
7	V2.conv	Conv2d	[1, 64, 28, 28]	[1, 128, 28, 28]	73856	True
8	V2.nonlin	ReLU	[1, 128, 28, 28]	[1, 128, 28, 28]	-	-
9	V2.pool	MaxPool2d	[1, 128, 28, 28]	[1, 128, 14, 14]	-	-
10	V2.output	Identity	[1, 128, 14, 14]	[1, 128, 14, 14]	-	-
12	V4.conv	Conv2d	[1, 128, 14, 14]	[1, 256, 14, 14]	295168	True
13	V4.nonlin	ReLU	[1, 256, 14, 14]	[1, 256, 14, 14]	-	-
14	V4.pool	MaxPool2d	[1, 256, 14, 14]	[1, 256, 7, 7]	-	-
15	V4.output	Identity	[1, 256, 7, 7]	[1, 256, 7, 7]	-	-
17	IT.conv_input	Conv2d	[1, 256, 7, 7]	[1, 512, 7, 7]	1180160	True
18	IT.norm_input	GroupNorm	[1, 512, 7, 7]	[1, 512, 7, 7]	1024	True
19	IT.nonlin_input	ReLU	[1, 512, 7, 7]	[1, 512, 7, 7]	-	-
20	IT.conv1	Conv2d	[1, 512, 7, 7]	[1, 512, 7, 7]	2359296	True
21	IT.norm1	GroupNorm	[1, 512, 7, 7]	[1, 512, 7, 7]	1024	True
22	IT.nonlin1	ReLU	[1, 512, 7, 7]	[1, 512, 7, 7]	-	-
23	IT.output	Identity	[1, 512, 7, 7]	[1, 512, 7, 7]	-	-
25	decoder.avgpool	AdaptiveAvgPool2d	[1, 512, 7, 7]	[1, 512, 1, 1]	-	-
26	decoder.flatten	Flatten	[1, 512, 1, 1]	[1, 512]	-	-
27	decoder.linear	Linear	[1, 512]	[1, 8]	4104	True
Total params: 3924104<br>Trainable params: 3924104<br>Non-trainable params: 0						

Table S4: Layer specific information about **C IT-IT** (CORnet IT-IT).

CL

Index	Name	Layer Type	Input Shape	Output Shape	Param #	Trainable
2	V1.conv_input	Conv2d	[1, 3, 224, 224]	[1, 64, 56, 56]	9472	True
3	V1.norm_input	GroupNorm	[1, 64, 56, 56]	[1, 64, 56, 56]	128	True
4	V1.nonlin_input	ReLU	[1, 64, 56, 56]	[1, 64, 56, 56]	-	-
5	V1.conv1	Conv2d	[1, 64, 56, 56]	[1, 64, 56, 56]	36864	True
6	V1.norm1	GroupNorm	[1, 64, 56, 56]	[1, 64, 56, 56]	128	True
7	V1.nonlin1	ReLU	[1, 64, 56, 56]	[1, 64, 56, 56]	-	-
8	V1.output	Identity	[1, 64, 56, 56]	[1, 64, 56, 56]	-	-
10	V2.conv_input	Conv2d	[1, 64, 56, 56]	[1, 128, 28, 28]	73856	True
11	V2.norm_input	GroupNorm	[1, 128, 28, 28]	[1, 128, 28, 28]	256	True
12	V2.nonlin_input	ReLU	[1, 128, 28, 28]	[1, 128, 28, 28]	-	-
13	V2.conv1	Conv2d	[1, 128, 28, 28]	[1, 128, 28, 28]	147456	True
14	V2.norm1	GroupNorm	[1, 128, 28, 28]	[1, 128, 28, 28]	256	True
15	V2.nonlin1	ReLU	[1, 128, 28, 28]	[1, 128, 28, 28]	-	-
16	V2.output	Identity	[1, 128, 28, 28]	[1, 128, 28, 28]	-	-
18	V4.conv_input	Conv2d	[1, 128, 28, 28]	[1, 256, 14, 14]	295168	True
19	V4.norm_input	GroupNorm	[1, 256, 14, 14]	[1, 256, 14, 14]	512	True
20	V4.nonlin_input	ReLU	[1, 256, 14, 14]	[1, 256, 14, 14]	-	-
21	V4.conv1	Conv2d	[1, 256, 14, 14]	[1, 256, 14, 14]	589824	True
22	V4.norm1	GroupNorm	[1, 256, 14, 14]	[1, 256, 14, 14]	512	True
23	V4.nonlin1	ReLU	[1, 256, 14, 14]	[1, 256, 14, 14]	-	-
24	V4.output	Identity	[1, 256, 14, 14]	[1, 256, 14, 14]	-	-
26	IT.conv_input	Conv2d	[1, 256, 14, 14]	[1, 512, 7, 7]	1180160	True
27	IT.norm_input	GroupNorm	[1, 512, 7, 7]	[1, 512, 7, 7]	1024	True
28	IT.nonlin_input	ReLU	[1, 512, 7, 7]	[1, 512, 7, 7]	-	-
29	IT.conv1	Conv2d	[1, 512, 7, 7]	[1, 512, 7, 7]	2359296	True
30	IT.norm1	GroupNorm	[1, 512, 7, 7]	[1, 512, 7, 7]	1024	True
31	IT.nonlin1	ReLU	[1, 512, 7, 7]	[1, 512, 7, 7]	-	-
32	IT.output	Identity	[1, 512, 7, 7]	[1, 512, 7, 7]	-	-
34	decoder.avgpool	AdaptiveAvgPool2d	[1, 512, 7, 7]	[1, 512, 1, 1]	-	-
35	decoder.flatten	Flatten	[1, 512, 1, 1]	[1, 512]	-	-
36	decoder.linear	Linear	[1, 512]	[1, 8]	4104	True
Total params: 4700040						
Trainable params: 4700040						
Non-trainable params: 0						

Table S5: Layer specific information about **CL**.

CT

Index	Name	Layer Type	Input Shape	Output Shape	Param #	Trainable
3	V1.ff_pass.0	Conv2d	[1, 3, 224, 224]	[1, 64, 56, 56]	9472	True
4	V1.ff_pass.1	GroupNorm	[1, 64, 56, 56]	[1, 64, 56, 56]	128	True
5	V1.ff_pass.2	ReLU	[1, 64, 56, 56]	[1, 64, 56, 56]	-	-
7	V1.td_pass.0	Conv2d	[1, 3, 224, 224]	[1, 64, 56, 56]	9472	True
8	V1.td_pass.1	GroupNorm	[1, 64, 56, 56]	[1, 64, 56, 56]	128	True
9	V1.td_pass.2	ReLU	[1, 64, 56, 56]	[1, 64, 56, 56]	-	-
11	V1.out_pass.0	Conv2d	[1, 128, 56, 56]	[1, 64, 56, 56]	401472	True
12	V1.out_pass.1	GroupNorm	[1, 64, 56, 56]	[1, 64, 56, 56]	128	True
13	V1.out_pass.2	ReLU	[1, 64, 56, 56]	[1, 64, 56, 56]	-	-
14	V1.output	Identity	[1, 64, 56, 56]	[1, 64, 56, 56]	-	-
17	V2.ff_pass.0	Conv2d	[1, 64, 56, 56]	[1, 128, 28, 28]	73856	True
18	V2.ff_pass.1	GroupNorm	[1, 128, 28, 28]	[1, 128, 28, 28]	256	True
19	V2.ff_pass.2	ReLU	[1, 128, 28, 28]	[1, 128, 28, 28]	-	-
21	V2.td_pass.0	Conv2d	[1, 2, 56, 56]	[1, 128, 28, 28]	2432	True
22	V2.td_pass.1	GroupNorm	[1, 128, 28, 28]	[1, 128, 28, 28]	256	True
23	V2.td_pass.2	ReLU	[1, 128, 28, 28]	[1, 128, 28, 28]	-	-
25	V2.out_pass.0	Conv2d	[1, 256, 28, 28]	[1, 128, 28, 28]	295040	True
26	V2.out_pass.1	GroupNorm	[1, 128, 28, 28]	[1, 128, 28, 28]	256	True
27	V2.out_pass.2	ReLU	[1, 128, 28, 28]	[1, 128, 28, 28]	-	-
28	V2.output	Identity	[1, 128, 28, 28]	[1, 128, 28, 28]	-	-
31	V4.ff_pass.0	Conv2d	[1, 128, 28, 28]	[1, 256, 14, 14]	295168	True
32	V4.ff_pass.1	GroupNorm	[1, 256, 14, 14]	[1, 256, 14, 14]	512	True
33	V4.ff_pass.2	ReLU	[1, 256, 14, 14]	[1, 256, 14, 14]	-	-
35	V4.td_pass.0	Conv2d	[1, 1, 28, 28]	[1, 256, 14, 14]	2560	True
36	V4.td_pass.1	GroupNorm	[1, 256, 14, 14]	[1, 256, 14, 14]	512	True
37	V4.td_pass.2	ReLU	[1, 256, 14, 14]	[1, 256, 14, 14]	-	-
39	V4.out_pass.0	Conv2d	[1, 512, 14, 14]	[1, 256, 14, 14]	1179904	True
40	V4.out_pass.1	GroupNorm	[1, 256, 14, 14]	[1, 256, 14, 14]	512	True
41	V4.out_pass.2	ReLU	[1, 256, 14, 14]	[1, 256, 14, 14]	-	-
42	V4.output	Identity	[1, 256, 14, 14]	[1, 256, 14, 14]	-	-
45	IT.ff_pass.0	Conv2d	[1, 256, 14, 14]	[1, 512, 7, 7]	1180160	True
46	IT.ff_pass.1	GroupNorm	[1, 512, 7, 7]	[1, 512, 7, 7]	1024	True
47	IT.ff_pass.2	ReLU	[1, 512, 7, 7]	[1, 512, 7, 7]	-	-
49	IT.td_pass.0	Conv2d	[1, 1, 14, 14]	[1, 512, 7, 7]	5120	True
50	IT.td_pass.1	GroupNorm	[1, 512, 7, 7]	[1, 512, 7, 7]	1024	True
51	IT.td_pass.2	ReLU	[1, 512, 7, 7]	[1, 512, 7, 7]	-	-
53	IT.out_pass.0	Conv2d	[1, 1024, 7, 7]	[1, 512, 7, 7]	4719104	True
54	IT.out_pass.1	GroupNorm	[1, 512, 7, 7]	[1, 512, 7, 7]	1024	True
55	IT.out_pass.2	ReLU	[1, 512, 7, 7]	[1, 512, 7, 7]	-	-
56	IT.output	Identity	[1, 512, 7, 7]	[1, 512, 7, 7]	-	-
58	decoder.avgpool	AdaptiveAvgPool2d	[1, 512, 7, 7]	[1, 512, 1, 1]	-	-
59	decoder.flatten	Flatten	[1, 512, 1, 1]	[1, 512]	-	-
60	decoder.linear	Linear	[1, 512]	[1, 8]	4104	True
Total params: 8183624<br>Trainable params: 8183624<br>Non-trainable params: 0						

Table S6: Layer specific information about **CT**.

CD

Index	Name	Layer Type	Input Shape	Output Shape	Param #	Trainable
3	V1.0.conv	Conv2d	[1, 3, 224, 224]	[1, 64, 112, 112]	9472	True
4	V1.0.nonlin	ReLU	[1, 64, 112, 112]	[1, 64, 112, 112]	-	-
5	V1.0.norm	BatchNorm2d	-	-	128	True
6	V1.0.output	Identity	[1, 64, 112, 112]	[1, 64, 112, 112]	-	-
8	V1.1.conv	Conv2d	[1, 64, 112, 112]	[1, 64, 112, 112]	36928	True
9	V1.1.nonlin	ReLU	[1, 64, 112, 112]	[1, 64, 112, 112]	-	-
10	V1.1.norm	BatchNorm2d	-	-	128	True
11	V1.1.output	Identity	[1, 64, 112, 112]	[1, 64, 112, 112]	-	-
13	V1.2.conv	Conv2d	[1, 64, 112, 112]	[1, 64, 112, 112]	36928	True
14	V1.2.nonlin	ReLU	[1, 64, 112, 112]	[1, 64, 112, 112]	-	-
15	V1.2.norm	BatchNorm2d	-	-	128	True
16	V1.2.output	Identity	[1, 64, 112, 112]	[1, 64, 112, 112]	-	-
18	V1.3.conv	Conv2d	[1, 64, 112, 112]	[1, 64, 112, 112]	36928	True
19	V1.3.nonlin	ReLU	[1, 64, 112, 112]	[1, 64, 112, 112]	-	-
20	V1.3.pool	MaxPool2d	[1, 64, 112, 112]	[1, 64, 56, 56]	-	-
21	V1.3.output	Identity	[1, 64, 56, 56]	[1, 64, 56, 56]	-	-
22	V1.4	BatchNorm2d	[1, 64, 56, 56]	[1, 64, 56, 56]	128	True
25	V2.0.conv	Conv2d	[1, 64, 56, 56]	[1, 128, 56, 56]	73856	True
26	V2.0.nonlin	ReLU	[1, 128, 56, 56]	[1, 128, 56, 56]	-	-
27	V2.0.norm	BatchNorm2d	-	-	256	True
28	V2.0.output	Identity	[1, 128, 56, 56]	[1, 128, 56, 56]	-	-
30	V2.1.conv	Conv2d	[1, 128, 56, 56]	[1, 128, 56, 56]	147584	True
31	V2.1.nonlin	ReLU	[1, 128, 56, 56]	[1, 128, 56, 56]	-	-
32	V2.1.norm	BatchNorm2d	-	-	256	True
33	V2.1.output	Identity	[1, 128, 56, 56]	[1, 128, 56, 56]	-	-
35	V2.2.conv	Conv2d	[1, 128, 56, 56]	[1, 128, 56, 56]	147584	True
36	V2.2.nonlin	ReLU	[1, 128, 56, 56]	[1, 128, 56, 56]	-	-
37	V2.2.norm	BatchNorm2d	-	-	256	True
38	V2.2.output	Identity	[1, 128, 56, 56]	[1, 128, 56, 56]	-	-
40	V2.3.conv	Conv2d	[1, 128, 56, 56]	[1, 128, 56, 56]	147584	True
41	V2.3.nonlin	ReLU	[1, 128, 56, 56]	[1, 128, 56, 56]	-	-
42	V2.3.pool	MaxPool2d	[1, 128, 56, 56]	[1, 128, 28, 28]	-	-
43	V2.3.output	Identity	[1, 128, 28, 28]	[1, 128, 28, 28]	-	-
44	V2.4	BatchNorm2d	[1, 128, 28, 28]	[1, 128, 28, 28]	256	True
47	V4.0.conv	Conv2d	[1, 128, 28, 28]	[1, 256, 28, 28]	295168	True
48	V4.0.nonlin	ReLU	[1, 256, 28, 28]	[1, 256, 28, 28]	-	-
49	V4.0.norm	BatchNorm2d	-	-	512	True
50	V4.0.output	Identity	[1, 256, 28, 28]	[1, 256, 28, 28]	-	-
52	V4.1.conv	Conv2d	[1, 256, 28, 28]	[1, 256, 28, 28]	590080	True
53	V4.1.nonlin	ReLU	[1, 256, 28, 28]	[1, 256, 28, 28]	-	-
54	V4.1.norm	BatchNorm2d	-	-	512	True
55	V4.1.output	Identity	[1, 256, 28, 28]	[1, 256, 28, 28]	-	-
57	V4.2.conv	Conv2d	[1, 256, 28, 28]	[1, 256, 28, 28]	590080	True
58	V4.2.nonlin	ReLU	[1, 256, 28, 28]	[1, 256, 28, 28]	-	-
59	V4.2.norm	BatchNorm2d	-	-	512	True
60	V4.2.output	Identity	[1, 256, 28, 28]	[1, 256, 28, 28]	-	-
62	V4.3.conv	Conv2d	[1, 256, 28, 28]	[1, 256, 28, 28]	590080	True
63	V4.3.nonlin	ReLU	[1, 256, 28, 28]	[1, 256, 28, 28]	-	-
64	V4.3.pool	MaxPool2d	[1, 256, 28, 28]	[1, 256, 14, 14]	-	-
65	V4.3.output	Identity	[1, 256, 14, 14]	[1, 256, 14, 14]	-	-
66	V4.4	BatchNorm2d	[1, 256, 14, 14]	[1, 256, 14, 14]	512	True
69	IT.0.conv	Conv2d	[1, 256, 14, 14]	[1, 512, 14, 14]	1180160	True
70	IT.0.nonlin	ReLU	[1, 512, 14, 14]	[1, 512, 14, 14]	-	-
71	IT.0.norm	BatchNorm2d	-	-	1024	True
72	IT.0.output	Identity	[1, 512, 14, 14]	[1, 512, 14, 14]	-	-
74	IT.1.conv	Conv2d	[1, 512, 14, 14]	[1, 512, 14, 14]	2359808	True
75	IT.1.nonlin	ReLU	[1, 512, 14, 14]	[1, 512, 14, 14]	-	-
76	IT.1.norm	BatchNorm2d	-	-	1024	True
77	IT.1.output	Identity	[1, 512, 14, 14]	[1, 512, 14, 14]	-	-
79	IT.2.conv	Conv2d	[1, 512, 14, 14]	[1, 512, 14, 14]	2359808	True
80	IT.2.nonlin	ReLU	[1, 512, 14, 14]	[1, 512, 14, 14]	-	-
81	IT.2.norm	BatchNorm2d	-	-	1024	True
82	IT.2.output	Identity	[1, 512, 14, 14]	[1, 512, 14, 14]	-	-
84	IT.3.conv	Conv2d	[1, 512, 14, 14]	[1, 512, 14, 14]	2359808	True
85	IT.3.nonlin	ReLU	[1, 512, 14, 14]	[1, 512, 14, 14]	-	-
86	IT.3.pool	MaxPool2d	[1, 512, 14, 14]	[1, 512, 7, 7]	-	-
87	IT.3.output	Identity	[1, 512, 7, 7]	[1, 512, 7, 7]	-	-
88	IT.4	BatchNorm2d	[1, 512, 7, 7]	[1, 512, 7, 7]	1024	True
90	decoder.avgpool	AdaptiveAvgPool2d	[1, 512, 7, 7]	[1, 512, 1, 1]	-	-
91	decoder.flatten	Flatten	[1, 512, 1, 1]	[1, 512]	-	-
92	decoder.linear	Linear	[1, 512]	[1, 8]	4104	True
93	decoder.output	Identity	[1, 8]	[1, 8]	-	-
Total params: 10973640						
Trainable params: 10973640						
Non-trainable params: 0						

Table S7: Layer-specific information about **CD**.

CLT

Index	Name	Layer Type	Input Shape	Output Shape	Param #	Trainable
3	V1.ff_pass.0	Conv2d	[1, 3, 224, 224]	[1, 64, 56, 56]	9472	True
4	V1.ff_pass.1	GroupNorm	[1, 64, 56, 56]	[1, 64, 56, 56]	128	True
5	V1.ff_pass.2	ReLU	[1, 64, 56, 56]	[1, 64, 56, 56]	-	-
7	V1.rr_pass.0	Conv2d	[1, 64, 56, 56]	[1, 64, 56, 56]	4160	True
8	V1.rr_pass.1	GroupNorm	[1, 64, 56, 56]	[1, 64, 56, 56]	128	True
9	V1.rr_pass.2	ReLU	[1, 64, 56, 56]	[1, 64, 56, 56]	-	-
10	V1.rr_pass.3	Dropout	[1, 64, 56, 56]	[1, 64, 56, 56]	-	-
12	V1.td_pass.0	Conv2d	[1, 3, 224, 224]	[1, 64, 56, 56]	9472	True
13	V1.td_pass.1	GroupNorm	[1, 64, 56, 56]	[1, 64, 56, 56]	128	True
14	V1.td_pass.2	ReLU	[1, 64, 56, 56]	[1, 64, 56, 56]	-	-
16	V1.out_pass.0	Conv2d	[1, 192, 56, 56]	[1, 64, 56, 56]	602176	True
17	V1.out_pass.1	GroupNorm	[1, 64, 56, 56]	[1, 64, 56, 56]	128	True
18	V1.out_pass.2	ReLU	[1, 64, 56, 56]	[1, 64, 56, 56]	-	-
19	V1.output	Identity	[1, 64, 56, 56]	[1, 64, 56, 56]	-	-
22	V2.ff_pass.0	Conv2d	[1, 64, 56, 56]	[1, 128, 28, 28]	73856	True
23	V2.ff_pass.1	GroupNorm	[1, 128, 28, 28]	[1, 128, 28, 28]	256	True
24	V2.ff_pass.2	ReLU	[1, 128, 28, 28]	[1, 128, 28, 28]	-	-
26	V2.rr_pass.0	Conv2d	[1, 128, 28, 28]	[1, 128, 28, 28]	16512	True
27	V2.rr_pass.1	GroupNorm	[1, 128, 28, 28]	[1, 128, 28, 28]	256	True
28	V2.rr_pass.2	ReLU	[1, 128, 28, 28]	[1, 128, 28, 28]	-	-
29	V2.rr_pass.3	Dropout	[1, 128, 28, 28]	[1, 128, 28, 28]	-	-
31	V2.td_pass.0	Conv2d	[1, 2, 56, 56]	[1, 128, 28, 28]	2432	True
32	V2.td_pass.1	GroupNorm	[1, 128, 28, 28]	[1, 128, 28, 28]	256	True
33	V2.td_pass.2	ReLU	[1, 128, 28, 28]	[1, 128, 28, 28]	-	-
35	V2.out_pass.0	Conv2d	[1, 384, 28, 28]	[1, 128, 28, 28]	442496	True
36	V2.out_pass.1	GroupNorm	[1, 128, 28, 28]	[1, 128, 28, 28]	256	True
37	V2.out_pass.2	ReLU	[1, 128, 28, 28]	[1, 128, 28, 28]	-	-
38	V2.output	Identity	[1, 128, 28, 28]	[1, 128, 28, 28]	-	-
41	V4.ff_pass.0	Conv2d	[1, 128, 28, 28]	[1, 256, 14, 14]	295168	True
42	V4.ff_pass.1	GroupNorm	[1, 256, 14, 14]	[1, 256, 14, 14]	512	True
43	V4.ff_pass.2	ReLU	[1, 256, 14, 14]	[1, 256, 14, 14]	-	-
45	V4.rr_pass.0	Conv2d	[1, 256, 14, 14]	[1, 256, 14, 14]	65792	True
46	V4.rr_pass.1	GroupNorm	[1, 256, 14, 14]	[1, 256, 14, 14]	512	True
47	V4.rr_pass.2	ReLU	[1, 256, 14, 14]	[1, 256, 14, 14]	-	-
48	V4.rr_pass.3	Dropout	[1, 256, 14, 14]	[1, 256, 14, 14]	-	-
50	V4.td_pass.0	Conv2d	[1, 1, 28, 28]	[1, 256, 14, 14]	2560	True
51	V4.td_pass.1	GroupNorm	[1, 256, 14, 14]	[1, 256, 14, 14]	512	True
52	V4.td_pass.2	ReLU	[1, 256, 14, 14]	[1, 256, 14, 14]	-	-
54	V4.out_pass.0	Conv2d	[1, 768, 14, 14]	[1, 256, 14, 14]	1769728	True
55	V4.out_pass.1	GroupNorm	[1, 256, 14, 14]	[1, 256, 14, 14]	512	True
56	V4.out_pass.2	ReLU	[1, 256, 14, 14]	[1, 256, 14, 14]	-	-
57	V4.output	Identity	[1, 256, 14, 14]	[1, 256, 14, 14]	-	-
60	IT.ff_pass.0	Conv2d	[1, 256, 14, 14]	[1, 512, 7, 7]	1180160	True
61	IT.ff_pass.1	GroupNorm	[1, 512, 7, 7]	[1, 512, 7, 7]	1024	True
62	IT.ff_pass.2	ReLU	[1, 512, 7, 7]	[1, 512, 7, 7]	-	-
64	IT.rr_pass.0	Conv2d	[1, 512, 7, 7]	[1, 512, 7, 7]	262656	True
65	IT.rr_pass.1	GroupNorm	[1, 512, 7, 7]	[1, 512, 7, 7]	1024	True
66	IT.rr_pass.2	ReLU	[1, 512, 7, 7]	[1, 512, 7, 7]	-	-
67	IT.rr_pass.3	Dropout	[1, 512, 7, 7]	[1, 512, 7, 7]	-	-
69	IT.td_pass.0	Conv2d	[1, 1, 14, 14]	[1, 512, 7, 7]	5120	True
70	IT.td_pass.1	GroupNorm	[1, 512, 7, 7]	[1, 512, 7, 7]	1024	True
71	IT.td_pass.2	ReLU	[1, 512, 7, 7]	[1, 512, 7, 7]	-	-
73	IT.out_pass.0	Conv2d	[1, 1536, 7, 7]	[1, 512, 7, 7]	7078400	True
74	IT.out_pass.1	GroupNorm	[1, 512, 7, 7]	[1, 512, 7, 7]	1024	True
75	IT.out_pass.2	ReLU	[1, 512, 7, 7]	[1, 512, 7, 7]	-	-
76	IT.output	Identity	[1, 512, 7, 7]	[1, 512, 7, 7]	-	-
78	decoder.avgpool	AdaptiveAvgPool2d	[1, 512, 7, 7]	[1, 512, 1, 1]	-	-
79	decoder.flatten	Flatten	[1, 512, 1, 1]	[1, 512]	-	-
80	decoder.linear	Linear	[1, 512]	[1, 8]	4104	True
Total params: 11831944						
Trainable params: 11831944						
Non-trainable params: 0						

Table S8: Layer-specific information about **CLT**.

CS

Index	Name	Layer Type	Input Shape	Output Shape	Param #	Trainable
2	V1.conv1	Conv2d	[1, 3, 224, 224]	[1, 64, 112, 112]	9408	True
3	V1.norm1	BatchNorm2d	[1, 64, 112, 112]	[1, 64, 112, 112]	128	True
4	V1.nonlin1	ReLU	[1, 64, 112, 112]	[1, 64, 112, 112]	-	-
5	V1.pool	MaxPool2d	[1, 64, 112, 112]	[1, 64, 56, 56]	-	-
6	V1.conv2	Conv2d	[1, 64, 56, 56]	[1, 64, 56, 56]	36864	True
7	V1.norm2	BatchNorm2d	[1, 64, 56, 56]	[1, 64, 56, 56]	128	True
8	V1.nonlin2	ReLU	[1, 64, 56, 56]	[1, 64, 56, 56]	-	-
9	V1.output	Identity	[1, 64, 56, 56]	[1, 64, 56, 56]	-	-
11	V2.conv_input	Conv2d	[1, 64, 56, 56]	[1, 128, 56, 56]	8192	True
12	V2.skip	Conv2d	[1, 128, 56, 56]	[1, 128, 28, 28]	16384	True
13	V2.norm_skip	BatchNorm2d	[1, 128, 28, 28]	[1, 128, 28, 28]	256	True
14	V2.conv1	Conv2d	[1, 128, 28, 28]	[1, 512, 28, 28]	65536	True
15	V2.nonlin1	ReLU	[1, 512, 28, 28]	[1, 512, 28, 28]	-	-
16	V2.conv2	Conv2d	[1, 512, 28, 28]	[1, 512, 28, 28]	2359296	True
17	V2.nonlin2	ReLU	[1, 512, 28, 28]	[1, 512, 28, 28]	-	-
18	V2.conv3	Conv2d	[1, 512, 28, 28]	[1, 128, 28, 28]	65536	True
19	V2.nonlin3	ReLU	[1, 128, 28, 28]	[1, 128, 28, 28]	-	-
20	V2.output	Identity	[1, 128, 28, 28]	[1, 128, 28, 28]	-	-
21	V2.norm1_0	BatchNorm2d	[1, 512, 56, 56]	[1, 512, 56, 56]	1024	True
22	V2.norm2_0	BatchNorm2d	[1, 512, 28, 28]	[1, 512, 28, 28]	1024	True
23	V2.norm3_0	BatchNorm2d	[1, 128, 28, 28]	[1, 128, 28, 28]	256	True
24	V2.norm1_1	BatchNorm2d	[1, 512, 28, 28]	[1, 512, 28, 28]	1024	True
25	V2.norm2_1	BatchNorm2d	[1, 512, 28, 28]	[1, 512, 28, 28]	1024	True
26	V2.norm3_1	BatchNorm2d	[1, 128, 28, 28]	[1, 128, 28, 28]	256	True
28	V4.conv_input	Conv2d	[1, 128, 28, 28]	[1, 256, 28, 28]	32768	True
29	V4.skip	Conv2d	[1, 256, 28, 28]	[1, 256, 14, 14]	65536	True
30	V4.norm_skip	BatchNorm2d	[1, 256, 14, 14]	[1, 256, 14, 14]	512	True
31	V4.conv1	Conv2d	[1, 256, 14, 14]	[1, 1024, 14, 14]	262144	True
32	V4.nonlin1	ReLU	[1, 1024, 14, 14]	[1, 1024, 14, 14]	-	-
33	V4.conv2	Conv2d	[1, 1024, 14, 14]	[1, 1024, 14, 14]	9437184	True
34	V4.nonlin2	ReLU	[1, 1024, 14, 14]	[1, 1024, 14, 14]	-	-
35	V4.conv3	Conv2d	[1, 1024, 14, 14]	[1, 256, 14, 14]	262144	True
36	V4.nonlin3	ReLU	[1, 256, 14, 14]	[1, 256, 14, 14]	-	-
37	V4.output	Identity	[1, 256, 14, 14]	[1, 256, 14, 14]	-	-
38	V4.norm1_0	BatchNorm2d	[1, 1024, 28, 28]	[1, 1024, 28, 28]	2048	True
39	V4.norm2_0	BatchNorm2d	[1, 1024, 14, 14]	[1, 1024, 14, 14]	2048	True
40	V4.norm3_0	BatchNorm2d	[1, 256, 14, 14]	[1, 256, 14, 14]	512	True
41	V4.norm1_1	BatchNorm2d	[1, 1024, 14, 14]	[1, 1024, 14, 14]	2048	True
42	V4.norm2_1	BatchNorm2d	[1, 1024, 14, 14]	[1, 1024, 14, 14]	2048	True
43	V4.norm3_1	BatchNorm2d	[1, 256, 14, 14]	[1, 256, 14, 14]	512	True
44	V4.norm1_2	BatchNorm2d	[1, 1024, 14, 14]	[1, 1024, 14, 14]	2048	True
45	V4.norm2_2	BatchNorm2d	[1, 1024, 14, 14]	[1, 1024, 14, 14]	2048	True
46	V4.norm3_2	BatchNorm2d	[1, 256, 14, 14]	[1, 256, 14, 14]	512	True
47	V4.norm1_3	BatchNorm2d	[1, 1024, 14, 14]	[1, 1024, 14, 14]	2048	True
48	V4.norm2_3	BatchNorm2d	[1, 1024, 14, 14]	[1, 1024, 14, 14]	2048	True
49	V4.norm3_3	BatchNorm2d	[1, 256, 14, 14]	[1, 256, 14, 14]	512	True
51	IT.conv_input	Conv2d	[1, 256, 14, 14]	[1, 512, 14, 14]	131072	True
52	IT.skip	Conv2d	[1, 512, 14, 14]	[1, 512, 7, 7]	262144	True
53	IT.norm_skip	BatchNorm2d	[1, 512, 7, 7]	[1, 512, 7, 7]	1024	True
54	IT.conv1	Conv2d	[1, 512, 7, 7]	[1, 2048, 7, 7]	1048576	True
55	IT.nonlin1	ReLU	[1, 2048, 7, 7]	[1, 2048, 7, 7]	-	-
56	IT.conv2	Conv2d	[1, 2048, 7, 7]	[1, 2048, 7, 7]	37748736	True
57	IT.nonlin2	ReLU	[1, 2048, 7, 7]	[1, 2048, 7, 7]	-	-
58	IT.conv3	Conv2d	[1, 2048, 7, 7]	[1, 512, 7, 7]	1048576	True
59	IT.nonlin3	ReLU	[1, 512, 7, 7]	[1, 512, 7, 7]	-	-
60	IT.output	Identity	[1, 512, 7, 7]	[1, 512, 7, 7]	-	-
61	IT.norm1_0	BatchNorm2d	[1, 2048, 14, 14]	[1, 2048, 14, 14]	4096	True
62	IT.norm2_0	BatchNorm2d	[1, 2048, 7, 7]	[1, 2048, 7, 7]	4096	True
63	IT.norm3_0	BatchNorm2d	[1, 512, 7, 7]	[1, 512, 7, 7]	1024	True
64	IT.norm1_1	BatchNorm2d	[1, 2048, 7, 7]	[1, 2048, 7, 7]	4096	True
65	IT.norm2_1	BatchNorm2d	[1, 2048, 7, 7]	[1, 2048, 7, 7]	4096	True
66	IT.norm3_1	BatchNorm2d	[1, 512, 7, 7]	[1, 512, 7, 7]	1024	True
68	decoder.avgpool	AdaptiveAvgPool2d	[1, 512, 7, 7]	[1, 512, 1, 1]	-	-
69	decoder.flatten	Flatten	[1, 512, 1, 1]	[1, 512]	-	-
70	decoder.linear	Linear	[1, 512]	[1, 8]	4104	True
71	decoder.output	Identity	[1, 8]	[1, 8]	-	-

Total params: 52907720
Trainable params: 52907720
Non-trainable params: 0

Table S9: Layer-specific information about **CS**.

B

Index	Name	Layer Type	Input Shape	Output Shape	Param #	Trainable
1	retina	Conv2d	[1, 3, 128, 128]	[1, 32, 128, 128]	2432	True
3	v1.bottomup	Conv2d	[1, 32, 128, 128]	[1, 64, 128, 128]	51200	True
4	v1_ln	LayerNorm	[1, 64, 128, 128]	[1, 64, 128, 128]	2097152	True
6	v2.bottom_up	Conv2d	[1, 64, 64, 64]	[1, 128, 64, 64]	204800	True
7	v2.pool	MaxPool2d	[1, 64, 128, 128]	[1, 64, 64, 64]	-	-
8	v2_ln	LayerNorm	[1, 128, 64, 64]	[1, 128, 64, 64]	1048576	True
10	v4.bottom_up	Conv2d	[1, 128, 32, 32]	[1, 256, 32, 32]	819200	True
11	v4.pool	MaxPool2d	[1, 128, 64, 64]	[1, 128, 32, 32]	-	-
12	v4_ln	LayerNorm	[1, 256, 32, 32]	[1, 256, 32, 32]	524288	True
14	it.bottom_up	Conv2d	[1, 256, 16, 16]	[1, 512, 16, 16]	3276800	True
15	it.pool	MaxPool2d	[1, 256, 32, 32]	[1, 256, 16, 16]	-	-
16	it_ln	LayerNorm	[1, 512, 16, 16]	[1, 512, 16, 16]	262144	True
17	gap	BLT_GAP	[1, 512, 16, 16]	[1, 512]	-	-
18	gap.pool	AvgPool2d	[1, 512, 16, 16]	[1, 512, 1, 1]	-	-
19	gap_ln	LayerNorm	[1, 512]	[1, 512]	1024	True
20	readout	Linear	[1, 512]	[1, 8]	4104	True
Total params: 11831944						
Trainable params: 11831944						
Non-trainable params: 0						

Table S10: Layer-specific information about **B**.

BL

Index	Name	Layer Type	Input Shape	Output Shape	Param #	Trainable
1	retina	Conv2d	[1, 3, 128, 128]	[1, 32, 128, 128]	2432	True
3	v1.bottom_up	Conv2d	[1, 32, 128, 128]	[1, 64, 128, 128]	51200	True
4	v1.lateral	Conv2d	-	-	102400	True
5	v1_ln	LayerNorm	[1, 64, 128, 128]	[1, 64, 128, 128]	2097152	True
7	v2.bottom_up	Conv2d	[1, 64, 64, 64]	[1, 128, 64, 64]	204800	True
8	v2.lateral	Conv2d	[1, 128, 64, 64]	[1, 128, 64, 64]	409600	True
9	v2.pool	MaxPool2d	[1, 64, 128, 128]	[1, 64, 64, 64]	-	-
10	v2_ln	LayerNorm	[1, 128, 64, 64]	[1, 128, 64, 64]	1048576	True
12	v4.bottom_up	Conv2d	[1, 128, 32, 32]	[1, 256, 32, 32]	819200	True
13	v4.lateral	Conv2d	[1, 256, 32, 32]	[1, 256, 32, 32]	1638400	True
14	v4.pool	MaxPool2d	[1, 128, 64, 64]	[1, 128, 32, 32]	-	-
15	v4_ln	LayerNorm	[1, 256, 32, 32]	[1, 256, 32, 32]	524288	True
17	it.bottom_up	Conv2d	[1, 256, 16, 16]	[1, 512, 16, 16]	3276800	True
18	it.lateral	Conv2d	[1, 512, 16, 16]	[1, 512, 16, 16]	6553600	True
19	it.pool	MaxPool2d	[1, 256, 32, 32]	[1, 256, 16, 16]	-	-
20	it_ln	LayerNorm	[1, 512, 16, 16]	[1, 512, 16, 16]	262144	True
21	gap	BLT_GAP	[1, 512, 16, 16]	[1, 512]	262144	True
22	gap.lateral	Linear	[1, 512]	[1, 512]	262144	True
23	gap.pool	AvgPool2d	[1, 512, 16, 16]	[1, 512, 1, 1]	-	-
24	gap_ln	LayerNorm	[1, 512]	[1, 512]	1024	True
25	readout	Linear	[1, 512]	[1, 8]	4104	True
Total params: 17520008						
Trainable params: 17520008						
Non-trainable params: 0						

Table S11: Layer-specific information about **BL**.

BT

Index	Name	Layer Type	Input Shape	Output Shape	Param #	Trainable
1	retina	Conv2d	[1, 3, 128, 128]	[1, 32, 128, 128]	2432	True
3	v1.bottom _up	Conv2d	[1, 32, 128, 128]	[1, 64, 128, 128]	51200	True
4	v1.top _down	ConvTranspose2d	-	-	204800	True
5	v1 _ln	LayerNorm	[1, 64, 128, 128]	[1, 64, 128, 128]	2097152	True
7	v2.bottom _up	Conv2d	[1, 64, 64, 64]	[1, 128, 64, 64]	204800	True
8	v2.top _down	ConvTranspose2d	[1, 256, 32, 32]	[1, 128, 64, 64]	819200	True
9	v2.pool	MaxPool2d	[1, 64, 128, 128]	[1, 64, 64, 64]	-	-
10	v2 _ln	LayerNorm	[1, 128, 64, 64]	[1, 128, 64, 64]	1048576	True
12	v4.bottom _up	Conv2d	[1, 128, 32, 32]	[1, 256, 32, 32]	819200	True
13	v4.top _down	ConvTranspose2d	[1, 512, 16, 16]	[1, 256, 32, 32]	3276800	True
14	v4.pool	MaxPool2d	[1, 128, 64, 64]	[1, 128, 32, 32]	-	-
15	v4 _ln	LayerNorm	[1, 256, 32, 32]	[1, 256, 32, 32]	524288	True
17	it.bottom _up	Conv2d	[1, 256, 16, 16]	[1, 512, 16, 16]	3276800	True
18	it.pool	MaxPool2d	[1, 256, 32, 32]	[1, 256, 16, 16]	-	-
19	it _ln	LayerNorm	[1, 512, 16, 16]	[1, 512, 16, 16]	262144	True
20	gap	BLT _GAP	[1, 512, 16, 16]	[1, 512]	-	-
21	gap.pool	AvgPool2d	[1, 512, 16, 16]	[1, 512, 1, 1]	-	-
22	gap _ln	LayerNorm	[1, 512]	[1, 512]	1024	True
23	readout	Linear	[1, 512]	[1, 8]	4104	True

Total params: 12592520
Trainable params: 12592520
Non-trainable params: 0

Table S12: Layer-specific information about **BT**.

BD

Index	Name	Layer Type	Input Shape	Output Shape	Param #	Trainable
1	retina	Conv2d	[1, 3, 128, 128]	[1, 32, 128, 128]	2432	True
4	v1.0.bottom_up	Conv2d	[1, 32, 128, 128]	[1, 64, 128, 128]	51200	True
6	v1.1.bottom_up	Conv2d	[1, 64, 128, 128]	[1, 64, 128, 128]	102400	True
8	v1.2.bottom_up	Conv2d	[1, 64, 128, 128]	[1, 64, 128, 128]	102400	True
9	v1_ln	LayerNorm	[1, 64, 128, 128]	[1, 64, 128, 128]	2097152	True
12	v2.0.bottom_up	Conv2d	[1, 64, 64, 64]	[1, 128, 64, 64]	204800	True
13	v2.0.pool	MaxPool2d	[1, 64, 128, 128]	[1, 64, 64, 64]	-	-
15	v2.1.bottom_up	Conv2d	[1, 128, 64, 64]	[1, 128, 64, 64]	409600	True
17	v2.2.bottom_up	Conv2d	[1, 128, 64, 64]	[1, 128, 64, 64]	409600	True
18	v2_ln	LayerNorm	[1, 128, 64, 64]	[1, 128, 64, 64]	1048576	True
21	v4.0.bottom_up	Conv2d	[1, 128, 32, 32]	[1, 256, 32, 32]	819200	True
22	v4.0.pool	MaxPool2d	[1, 128, 64, 64]	[1, 128, 32, 32]	-	-
24	v4.1.bottom_up	Conv2d	[1, 256, 32, 32]	[1, 256, 32, 32]	1638400	True
26	v4.2.bottom_up	Conv2d	[1, 256, 32, 32]	[1, 256, 32, 32]	1638400	True
27	v4_ln	LayerNorm	[1, 256, 32, 32]	[1, 256, 32, 32]	524288	True
30	it.0.bottom_up	Conv2d	[1, 256, 16, 16]	[1, 512, 16, 16]	3276800	True
31	it.0.pool	MaxPool2d	[1, 256, 32, 32]	[1, 256, 16, 16]	-	-
33	it.1.bottom_up	Conv2d	[1, 512, 16, 16]	[1, 512, 16, 16]	6553600	True
35	it.2.bottom_up	Conv2d	[1, 512, 16, 16]	[1, 512, 16, 16]	6553600	True
36	it_ln	LayerNorm	[1, 512, 16, 16]	[1, 512, 16, 16]	262144	True
37	gap	BLT_GAP	[1, 512, 16, 16]	[1, 512]	-	-
38	gap.pool	AvgPool2d	[1, 512, 16, 16]	[1, 512, 1, 1]	-	-
39	gap_ln	LayerNorm	[1, 512]	[1, 512]	1024	True
40	readout	Linear	[1, 512]	[1, 8]	4104	True

Total params: 25699720
Trainable params: 25699720
Non-trainable params: 0

Table S13: Layer-specific information about **BD**.

BLT

Index	Name	Layer Type	Input Shape	Output Shape	Param #	Trainable
1	retina	Conv2d	[1, 3, 128, 128]	[1, 32, 128, 128]	2432	True
3	v1.bottom _up	Conv2d	[1, 32, 128, 128]	[1, 64, 128, 128]	51200	True
4	v1.lateral	Conv2d	-	-	102400	True
5	v1.top _down	ConvTranspose2d	-	-	204800	True
6	v1 _ln	LayerNorm	[1, 64, 128, 128]	[1, 64, 128, 128]	2097152	True
8	v2.bottom _up	Conv2d	[1, 64, 64, 64]	[1, 128, 64, 64]	204800	True
9	v2.lateral	Conv2d	[1, 128, 64, 64]	[1, 128, 64, 64]	409600	True
10	v2.top _down	ConvTranspose2d	[1, 256, 32, 32]	[1, 128, 64, 64]	819200	True
11	v2.pool	MaxPool2d	[1, 64, 128, 128]	[1, 64, 64, 64]	-	-
12	v2 _ln	LayerNorm	[1, 128, 64, 64]	[1, 128, 64, 64]	1048576	True
14	v4.bottom _up	Conv2d	[1, 128, 32, 32]	[1, 256, 32, 32]	819200	True
15	v4.lateral	Conv2d	[1, 256, 32, 32]	[1, 256, 32, 32]	1638400	True
16	v4.top _down	ConvTranspose2d	[1, 512, 16, 16]	[1, 256, 32, 32]	3276800	True
17	v4.pool	MaxPool2d	[1, 128, 64, 64]	[1, 128, 32, 32]	-	-
18	v4 _ln	LayerNorm	[1, 256, 32, 32]	[1, 256, 32, 32]	524288	True
20	it.bottom _up	Conv2d	[1, 256, 16, 16]	[1, 512, 16, 16]	3276800	True
21	it.lateral	Conv2d	[1, 512, 16, 16]	[1, 512, 16, 16]	6553600	True
22	it.pool	MaxPool2d	[1, 256, 32, 32]	[1, 256, 16, 16]	-	-
23	it _ln	LayerNorm	[1, 512, 16, 16]	[1, 512, 16, 16]	262144	True
24	gap	BLT _GAP	[1, 512, 16, 16]	[1, 512]	262144	True
25	gap.lateral	Linear	[1, 512]	[1, 512]	262144	True
26	gap.pool	AvgPool2d	[1, 512, 16, 16]	[1, 512, 1, 1]	-	-
27	gap _ln	LayerNorm	[1, 512]	[1, 512]	1024	True
28	readout	Linear	[1, 512]	[1, 8]	4104	True
Total params: 21820808<br>Trainable params: 21820808<br>Non-trainable params: 0						

Table S14: Layer-specific information about **BLT**.

VGG11

Index	Name	Layer Type	Input Shape	Output Shape	Param #	Trainable
2	features.0	Conv2d	[1, 3, 224, 224]	[1, 64, 224, 224]	1792	True
3	features.1	BatchNorm2d	[1, 64, 224, 224]	[1, 64, 224, 224]	128	True
4	features.2	ReLU	[1, 64, 224, 224]	[1, 64, 224, 224]	-	-
5	features.3	MaxPool2d	[1, 64, 224, 224]	[1, 64, 112, 112]	-	-
6	features.4	Conv2d	[1, 64, 112, 112]	[1, 128, 112, 112]	73856	True
7	features.5	BatchNorm2d	[1, 128, 112, 112]	[1, 128, 112, 112]	256	True
8	features.6	ReLU	[1, 128, 112, 112]	[1, 128, 112, 112]	-	-
9	features.7	MaxPool2d	[1, 128, 112, 112]	[1, 128, 56, 56]	-	-
10	features.8	Conv2d	[1, 128, 56, 56]	[1, 256, 56, 56]	295168	True
11	features.9	BatchNorm2d	[1, 256, 56, 56]	[1, 256, 56, 56]	512	True
12	features.10	ReLU	[1, 256, 56, 56]	[1, 256, 56, 56]	-	-
13	features.11	Conv2d	[1, 256, 56, 56]	[1, 256, 56, 56]	590080	True
14	features.12	BatchNorm2d	[1, 256, 56, 56]	[1, 256, 56, 56]	512	True
15	features.13	ReLU	[1, 256, 56, 56]	[1, 256, 56, 56]	-	-
16	features.14	MaxPool2d	[1, 256, 56, 56]	[1, 256, 28, 28]	-	-
17	features.15	Conv2d	[1, 256, 28, 28]	[1, 512, 28, 28]	1180160	True
18	features.16	BatchNorm2d	[1, 512, 28, 28]	[1, 512, 28, 28]	1024	True
19	features.17	ReLU	[1, 512, 28, 28]	[1, 512, 28, 28]	-	-
20	features.18	Conv2d	[1, 512, 28, 28]	[1, 512, 28, 28]	2359808	True
21	features.19	BatchNorm2d	[1, 512, 28, 28]	[1, 512, 28, 28]	1024	True
22	features.20	ReLU	[1, 512, 28, 28]	[1, 512, 28, 28]	-	-
23	features.21	MaxPool2d	[1, 512, 28, 28]	[1, 512, 14, 14]	-	-
24	features.22	Conv2d	[1, 512, 14, 14]	[1, 512, 14, 14]	2359808	True
25	features.23	BatchNorm2d	[1, 512, 14, 14]	[1, 512, 14, 14]	1024	True
26	features.24	ReLU	[1, 512, 14, 14]	[1, 512, 14, 14]	-	-
27	features.25	Conv2d	[1, 512, 14, 14]	[1, 512, 14, 14]	2359808	True
28	features.26	BatchNorm2d	[1, 512, 14, 14]	[1, 512, 14, 14]	1024	True
29	features.27	ReLU	[1, 512, 14, 14]	[1, 512, 14, 14]	-	-
30	features.28	MaxPool2d	[1, 512, 14, 14]	[1, 512, 7, 7]	-	-
31	classifier	Linear	[1, 25088]	[1, 8]	200712	True
Total params: 9426696						
Trainable params: 9426696						
Non-trainable params: 0						

Table S15: Layer-specific information about **VGG11**.

VGG16

Index	Name	Layer Type	Input Shape	Output Shape	Param #	Trainable
2	features.0	Conv2d	[1, 3, 224, 224]	[1, 64, 224, 224]	1792	True
3	features.1	BatchNorm2d	[1, 64, 224, 224]	[1, 64, 224, 224]	128	True
4	features.2	ReLU	[1, 64, 224, 224]	[1, 64, 224, 224]	-	-
5	features.3	Conv2d	[1, 64, 224, 224]	[1, 64, 224, 224]	36928	True
6	features.4	BatchNorm2d	[1, 64, 224, 224]	[1, 64, 224, 224]	128	True
7	features.5	ReLU	[1, 64, 224, 224]	[1, 64, 224, 224]	-	-
8	features.6	MaxPool2d	[1, 64, 224, 224]	[1, 64, 112, 112]	-	-
9	features.7	Conv2d	[1, 64, 112, 112]	[1, 128, 112, 112]	73856	True
10	features.8	BatchNorm2d	[1, 128, 112, 112]	[1, 128, 112, 112]	256	True
11	features.9	ReLU	[1, 128, 112, 112]	[1, 128, 112, 112]	-	-
12	features.10	Conv2d	[1, 128, 112, 112]	[1, 128, 112, 112]	147584	True
13	features.11	BatchNorm2d	[1, 128, 112, 112]	[1, 128, 112, 112]	256	True
14	features.12	ReLU	[1, 128, 112, 112]	[1, 128, 112, 112]	-	-
15	features.13	MaxPool2d	[1, 128, 112, 112]	[1, 128, 56, 56]	-	-
16	features.14	Conv2d	[1, 128, 56, 56]	[1, 256, 56, 56]	295168	True
17	features.15	BatchNorm2d	[1, 256, 56, 56]	[1, 256, 56, 56]	512	True
18	features.16	ReLU	[1, 256, 56, 56]	[1, 256, 56, 56]	-	-
19	features.17	Conv2d	[1, 256, 56, 56]	[1, 256, 56, 56]	590080	True
20	features.18	BatchNorm2d	[1, 256, 56, 56]	[1, 256, 56, 56]	512	True
21	features.19	ReLU	[1, 256, 56, 56]	[1, 256, 56, 56]	-	-
22	features.20	Conv2d	[1, 256, 56, 56]	[1, 256, 56, 56]	590080	True
23	features.21	BatchNorm2d	[1, 256, 56, 56]	[1, 256, 56, 56]	512	True
24	features.22	ReLU	[1, 256, 56, 56]	[1, 256, 56, 56]	-	-
25	features.23	MaxPool2d	[1, 256, 56, 56]	[1, 256, 28, 28]	-	-
26	features.24	Conv2d	[1, 256, 28, 28]	[1, 512, 28, 28]	1180160	True
27	features.25	BatchNorm2d	[1, 512, 28, 28]	[1, 512, 28, 28]	1024	True
28	features.26	ReLU	[1, 512, 28, 28]	[1, 512, 28, 28]	-	-
29	features.27	Conv2d	[1, 512, 28, 28]	[1, 512, 28, 28]	2359808	True
30	features.28	BatchNorm2d	[1, 512, 28, 28]	[1, 512, 28, 28]	1024	True
31	features.29	ReLU	[1, 512, 28, 28]	[1, 512, 28, 28]	-	-
32	features.30	Conv2d	[1, 512, 28, 28]	[1, 512, 28, 28]	2359808	True
33	features.31	BatchNorm2d	[1, 512, 28, 28]	[1, 512, 28, 28]	1024	True
34	features.32	ReLU	[1, 512, 28, 28]	[1, 512, 28, 28]	-	-
35	features.33	MaxPool2d	[1, 512, 28, 28]	[1, 512, 14, 14]	-	-
36	features.34	Conv2d	[1, 512, 14, 14]	[1, 512, 14, 14]	2359808	True
37	features.35	BatchNorm2d	[1, 512, 14, 14]	[1, 512, 14, 14]	1024	True
38	features.36	ReLU	[1, 512, 14, 14]	[1, 512, 14, 14]	-	-
39	features.37	Conv2d	[1, 512, 14, 14]	[1, 512, 14, 14]	2359808	True
40	features.38	BatchNorm2d	[1, 512, 14, 14]	[1, 512, 14, 14]	1024	True
41	features.39	ReLU	[1, 512, 14, 14]	[1, 512, 14, 14]	-	-
42	features.40	Conv2d	[1, 512, 14, 14]	[1, 512, 14, 14]	2359808	True
43	features.41	BatchNorm2d	[1, 512, 14, 14]	[1, 512, 14, 14]	1024	True
44	features.42	ReLU	[1, 512, 14, 14]	[1, 512, 14, 14]	-	-
45	features.43	MaxPool2d	[1, 512, 14, 14]	[1, 512, 7, 7]	-	-
47	classifier.0	Linear	[1, 25088]	[1, 4096]	102764544	True
48	classifier.1	ReLU	[1, 4096]	[1, 4096]	-	-
49	classifier.2	Dropout	[1, 4096]	[1, 4096]	-	-
50	classifier.3	Linear	[1, 4096]	[1, 4096]	16781312	True
51	classifier.4	ReLU	[1, 4096]	[1, 4096]	-	-
52	classifier.5	Dropout	[1, 4096]	[1, 4096]	-	-
53	classifier.6	Linear	[1, 4096]	[1, 8]	32776	True
Total params: 134301768						
Trainable params: 134301768						
Non-trainable params: 0						

Table S16: Layer-specific information about **VGG16**.

2 Model performance table

Table S17 below summarises the data used to make figure 6 of the article. It contains the average accuracy per task, for all models as well as for humans.

	Control	Light clutter	Heavy clutter	High pass	Low pass	Large deletion easy	Large deletion hard	Large apertures easy	Large apertures hard
CORnet_Z	0.925	0.831	0.644	0.48	0.229	0.871	0.674	0.799	0.455
CORnet_V1_V1	0.923	0.84	0.725	0.333	0.262	0.876	0.701	0.804	0.489
CORnet_IT_IT	0.95	0.895	0.794	0.5	0.212	0.913	0.762	0.758	0.395
CORnet_RT	0.964	0.906	0.857	0.493	0.324	0.938	0.771	0.853	0.476
CORnet_T	0.972	0.935	0.896	0.528	0.342	0.946	0.768	0.791	0.382
CORnet_LT	0.974	0.945	0.913	0.68	0.34	0.954	0.782	0.849	0.417
CORnet_ZD	0.984	0.957	0.9	0.7	0.3	0.968	0.807	0.895	0.569
CORnet_S	0.993	0.972	0.903	0.821	0.432	0.983	0.852	0.938	0.585
B_net	0.961	0.906	0.704	0.428	0.239	0.93	0.763	0.814	0.416
BL_net	0.981	0.961	0.9	0.753	0.339	0.961	0.822	0.808	0.362
BT_net	0.977	0.955	0.901	0.661	0.424	0.954	0.809	0.834	0.416
BD_net	0.963	0.924	0.778	0.71	0.293	0.939	0.793	0.827	0.425
BLT_net	0.984	0.965	0.928	0.773	0.404	0.963	0.818	0.815	0.374
VGG11	0.974	0.938	0.815	0.451	0.3	0.959	0.802	0.91	0.56
VGG16	0.991	0.974	0.907	0.684	0.339	0.987	0.902	0.919	0.595
Humans	0.969	0.963	0.963	0.965	0.653	0.958	0.899	0.938	0.807

	Large blobs hard	Large blobs easy	Small deletion easy	Small deletion hard	Small blobs easy	Small blobs hard	Small apertures easy	Small apertures hard
CORnet_Z	0.661	0.866	0.858	0.563	0.532	0.2	0.403	0.219
CORnet_V1_V1	0.71	0.868	0.851	0.572	0.492	0.223	0.439	0.22
CORnet_IT_IT	0.698	0.902	0.908	0.623	0.674	0.186	0.409	0.161
CORnet_RT	0.757	0.927	0.931	0.597	0.72	0.25	0.607	0.21
CORnet_T	0.7	0.925	0.939	0.633	0.818	0.273	0.578	0.169
CORnet_LT	0.715	0.929	0.952	0.639	0.747	0.2	0.545	0.173
CORnet_ZD	0.819	0.956	0.958	0.68	0.746	0.282	0.57	0.193
CORnet_S	0.847	0.972	0.979	0.76	0.769	0.305	0.54	0.156
B_net	0.719	0.918	0.929	0.641	0.596	0.224	0.403	0.169
BL_net	0.63	0.904	0.963	0.676	0.701	0.199	0.416	0.136
BT_net	0.696	0.905	0.946	0.63	0.741	0.219	0.526	0.155
BD_net	0.732	0.924	0.926	0.625	0.623	0.226	0.438	0.144
BLT_net	0.735	0.933	0.961	0.657	0.726	0.223	0.42	0.149
VGG11	0.812	0.956	0.96	0.719	0.78	0.286	0.562	0.261
VGG16	0.885	0.981	0.981	0.769	0.863	0.438	0.66	0.239
Humans	0.91	0.956	0.963	0.879	0.955	0.91	0.904	0.588

Table S17: Average performance per task per model.

3 Supplementary figures

3.1 Image manipulations detail

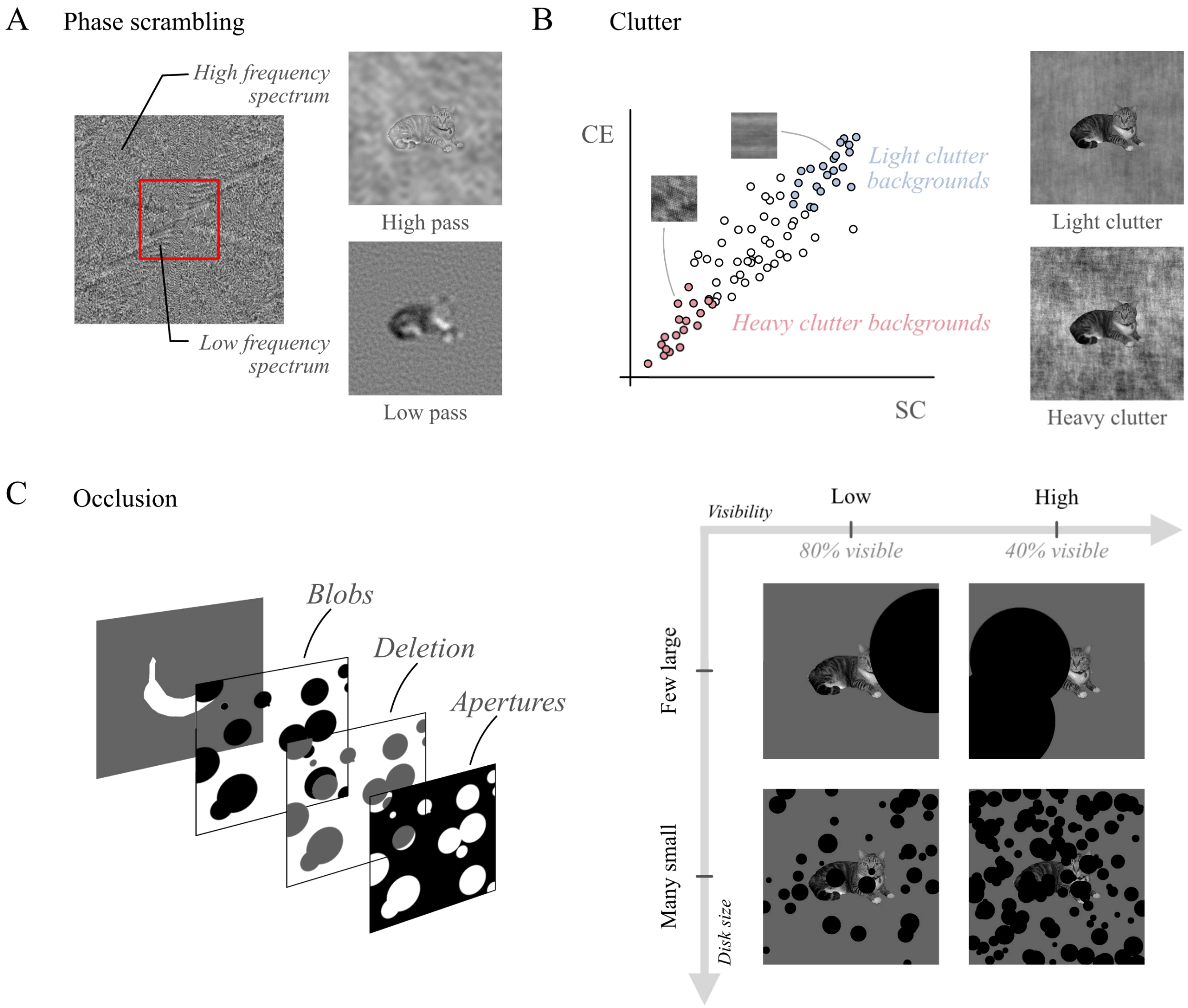

Figure S1: **Stimulus manipulations**. The stimulus set included eight object categories of 10 objects each, passed through a total of 16 visual manipulations. Objects in the images were rendered challenging by applying either *phase scrambling*, *clutter*, or *occlusion*. (A) Phase scrambling was applied by replacing the phase spectrum of images with random noise, on either sides of a 1.5 cpd threshold (red square not actual size). (B) Clutter was created following the method described in [**?**], whereby phase-scrambled versions of natural scenes taken from the MS COCO dataset were ranked on their *spatial coherence* and *contrast energy* values. Subsets of the most and less cluttered of a large number of such images were taken as light- and heavy-cluttered backgrounds for the stimuli. (C) Images were lightly or heavily occluded (percentage of object left visible: 80% and 40%, respectively), using many small or a few large disks. This was done in one of three possible fashions: adding black blobs, adding blobs the colour of the background (deletion) adding a full black occluder with disk-like apertures.

3.2 ImageNet training curves

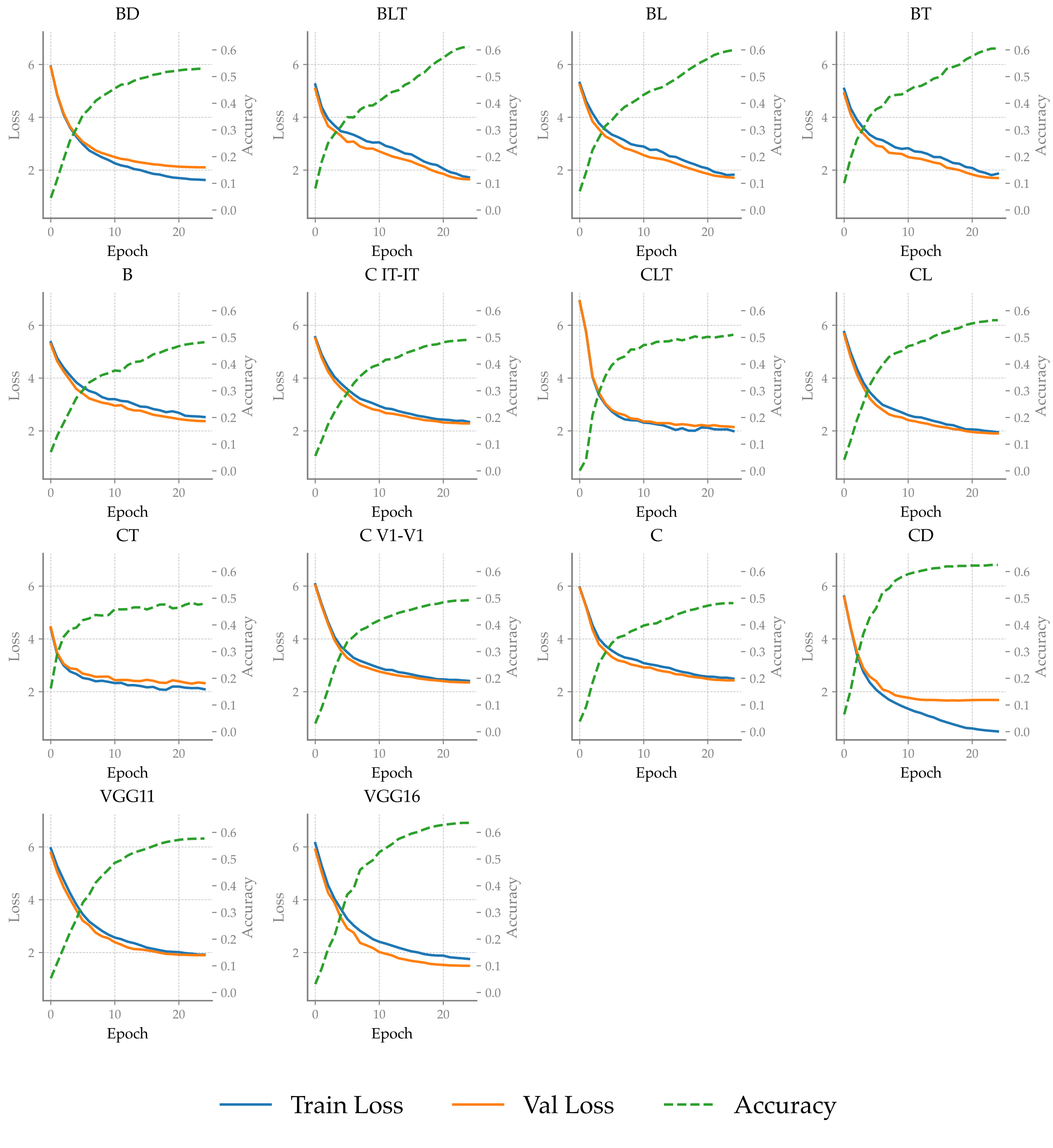

Figure S2: Training curves of all models trained in-house on ImageNet (i.e. all of them, except for CS, for which we used the pre-trained, publicly available version).

3.3 Fine-tuning validation accuracy

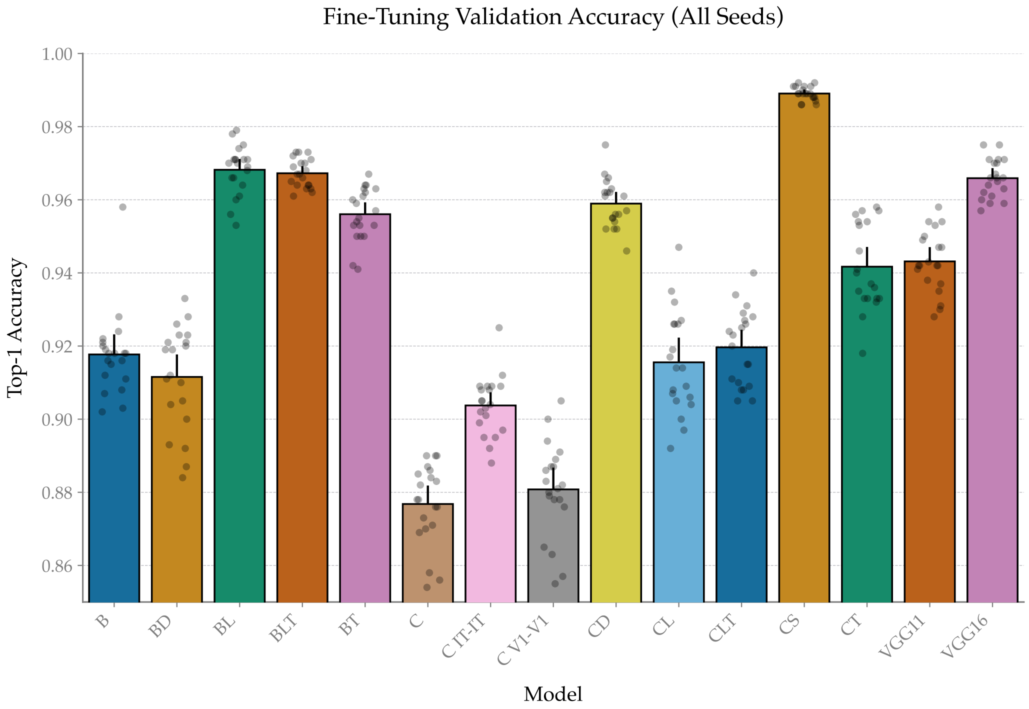

Figure S3: Accuracy per model in the validation of the fine-tuning on our eight categories.

3.4 Colour-blind friendly performance point plot

Supplementary figure S4 reproduces figure 6 in a less colour-heavy fashion.

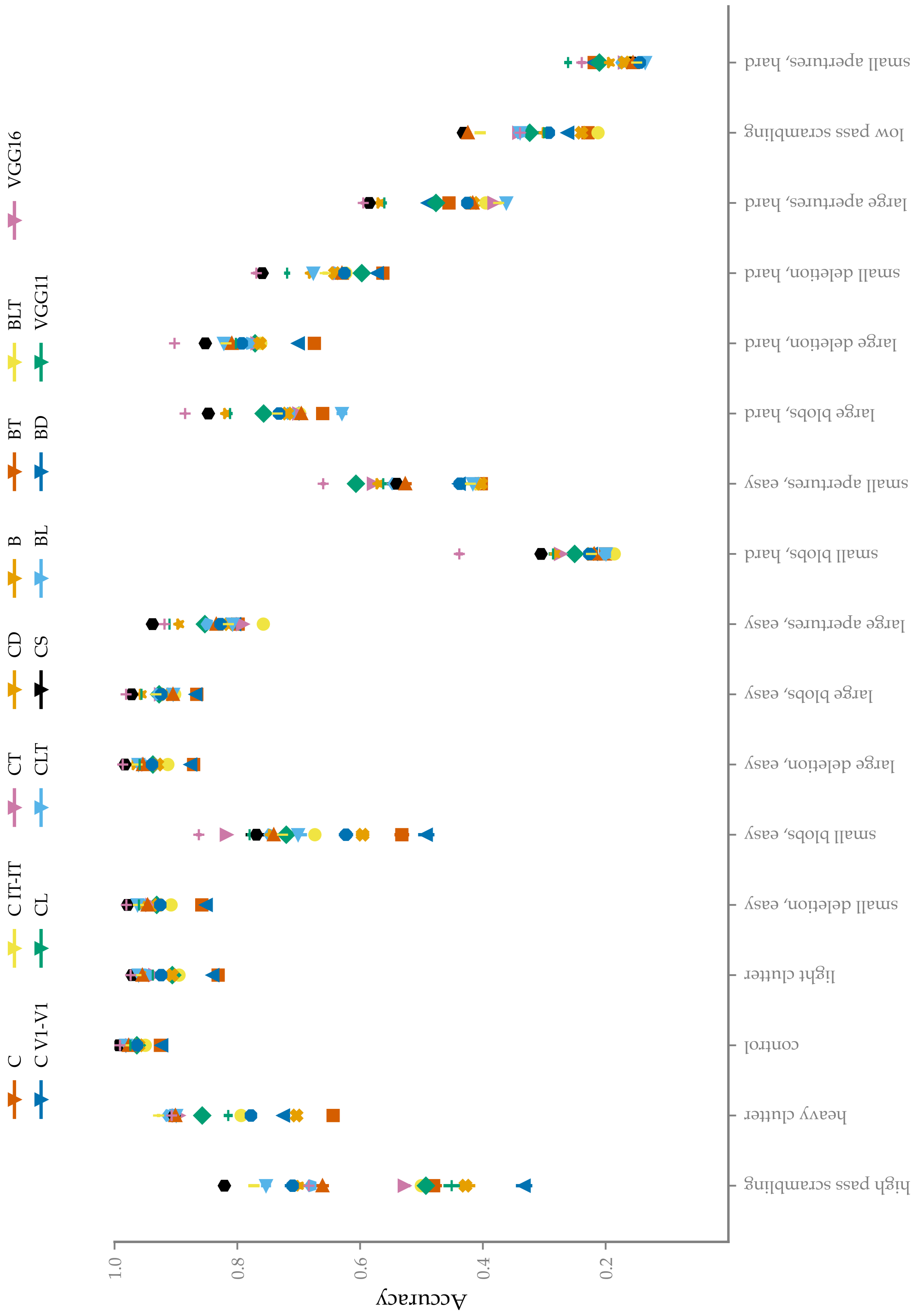

Figure S4: A more colour-blind friendly reproduction of figure 6. The colour palette has been adapted for more contrast. Shape distinctions have been added as well.

3.5 Confusion matrices

Supplementary figures S5 and S6 show the confusion matrices of human participants and of all the models used in this study, respectively. The human confusion matrix is made by summing up the confusion matrices of all participants. The confusion matrices of all models is made by summing up the confusion matrices of each initialisation seed.

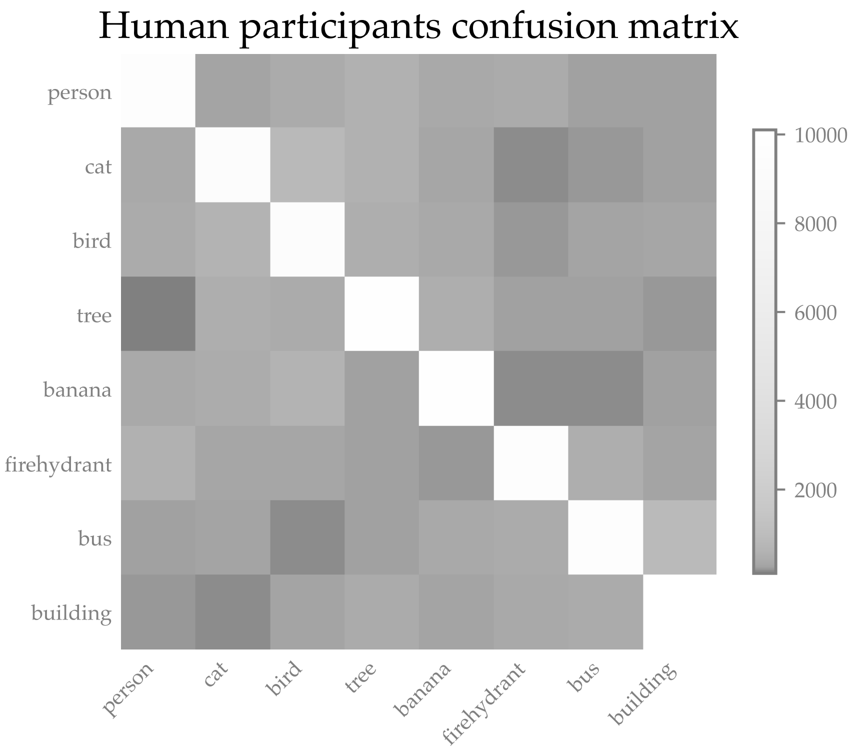

Figure S5: Confusion matrix of all human participants pooled together. Categories on the y axis are input images, categories on the x axis are the response categories.

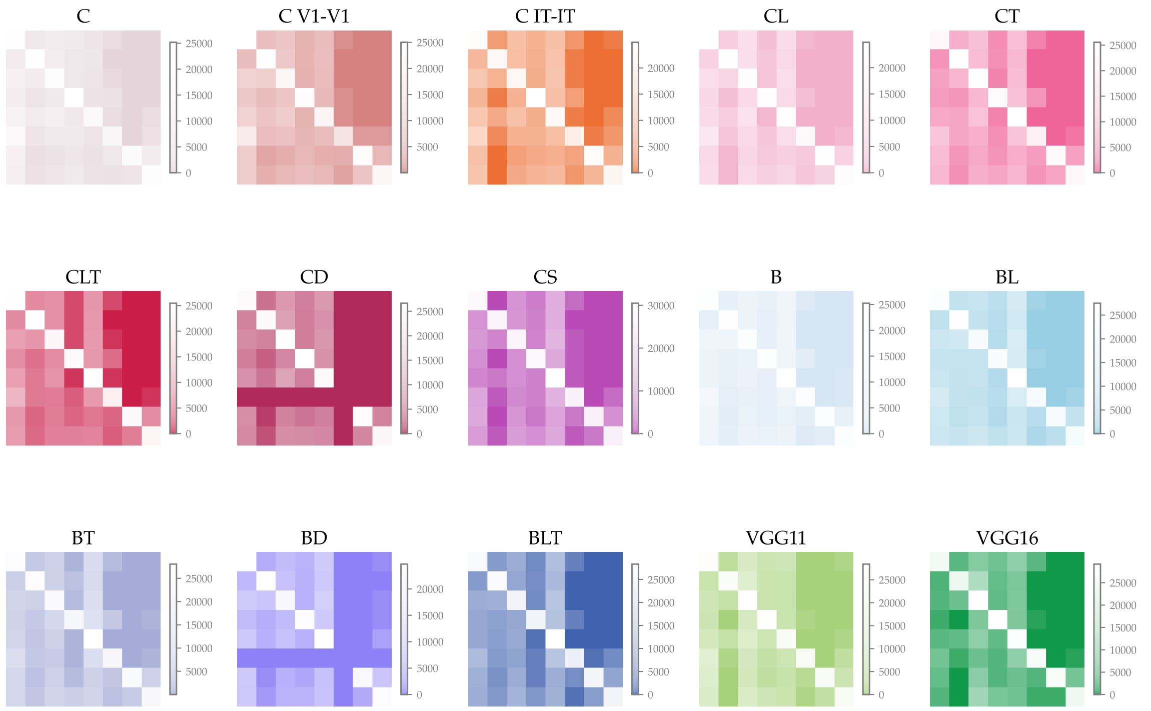

Figure S6: Confusion matrix of DNN models. Categories on the y axis are input images, categories on the x axis are the response categories. The order of categories is the same as in S5.

3.6 Participant-level confusion matrix correlation

We calculated participant-level correlations with model confusion matrices, taking a more conservative approach to confirm the disadvantage of adding recurrent connections to DNNs. We built a confusion matrix per subject (n=218) and correlated it with the confusion matrix of each model. With the correlation of each individual participant with each model, we calculated a 95% confidence interval around the mean, which confirmed our results.

This approach confirms results from the main text: CS and recurrent versions of B all have average participant-level correlation scores outside of and lower than the CI boundaries of the correlation scores of the larger feedforward models within their families (CD and BD), as well as their smaller, baseline counterparts. Additionally, all recurrent versions of C and B show correlations lower than VGG 16 (all below the CI lower boundary of 0.13). These values are striking when compared to model-model confusion matrix correlations: all models have a relatively higher average correlation with other models (Pearson's r, seed-level correlation with all other models in the range $0.24 - 0.94$, with average 0.73).

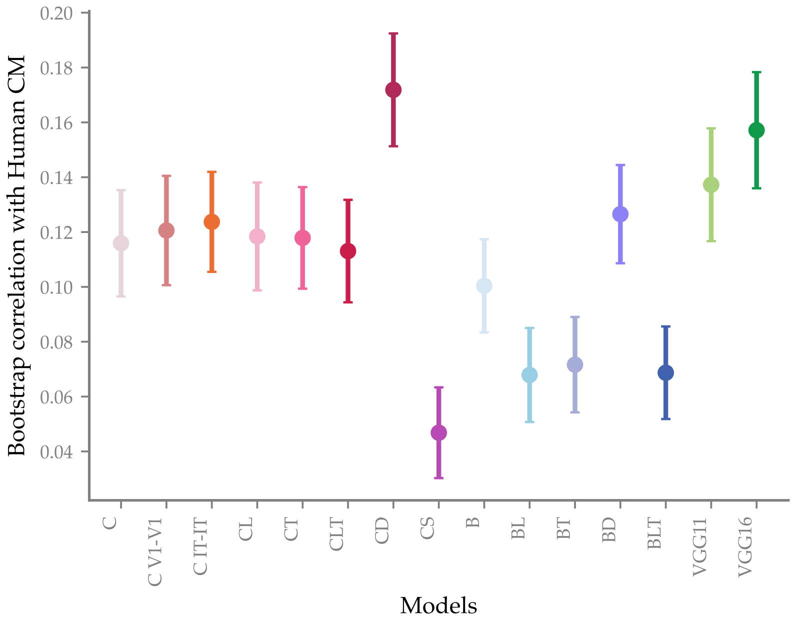

Figure S7: Average individual participant confusion matrix correlation with each model. Bars represent a 95% confidence interval around the mean. VGG 16 shows a higher correlation than any other model (all models below the lower CI boundary, 0.13)

3.7 Model-wise confusion matrix correlations

Supplementary figure S8 shows a matrix of correlations across models. Each square shows the average correlation value of 20 pairs of seed-level confusion matrices from two given models. Note the scale of the graph: correlations within models are mostly in the range of $0.3-1$. For reference, the confusion matrix correlations of models with humans range from 0.08 to 0.43. This indicates a larger agreement of models with each other than with human participants, sometimes in spite of architecture or model family. However, it is also noticeable that the range of within-model correlations sometimes overlaps with the range of correlations with humans. In particular, VGG16 seems to correlate better with humans (Pearson's r 0.43) than with the smaller C models (e.g. with C: 0.24, C V1-V1: 0.28).

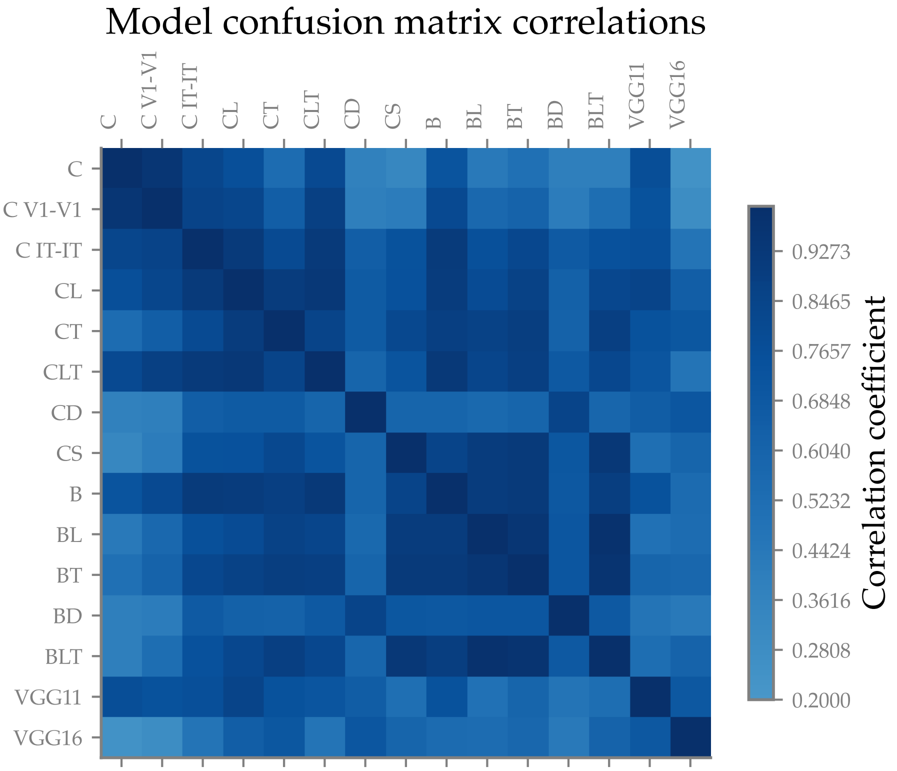

Figure S8: Correlation matrix: confusion matrices across models. Each cell represents the Pearson's r correlation between the confusion matrices of two models.
